# From loyalty to resignation: Patient–doctor figurations in type 1 diabetes

**DOI:** 10.1111/1467-9566.13304

**Published:** 2021-05-28

**Authors:** Alberto Ardissone

**Affiliations:** ^1^ Department of Law University of Macerata Macerata Italy

**Keywords:** diabetes, loyalty, patient–doctor figuration, power balance, relational sociology, resignation, self‐management, therapeutic devices

## Abstract

This paper contributes to the debate on the patient–doctor relationship by focussing on a specific chronic disease: type 1 diabetes. This field is characterised by an increasing use of technology, specifically therapeutic devices and a significant requirement of patient self‐management. This paper presents the main findings of research conducted in Italy in 2018. It is argued that this relationship is more properly described as an interdependent figuration of actors characterised by a dynamic process of power balances, which recalls Elias' (What is sociology? Columbia University Press, 1978) figurational‐processual and relational sociology. In this theoretical context, patients may manage their (dis)satisfaction with their diabetologists by choosing different behaviours that stem from Hirschman's archetype (Exit, voice, and loyalty. Responses to decline firms, organizations, and states. Harvard University Press, 1970): voice, exit, loyalty and, we would add, resignation. These categories are fluid, and all of them can be experienced by patients over time, depending on the quality of the figurations built among these transactors.

**Abbreviations**
Hgb: hemoglobin
INHS: Italian National Health Service
SAP: sensor‐augmented insulin pumps
TD: therapeutic device
T1D: Type 1 Diabetes
Yrs: years

## INTRODUCTION: DIABETES AND TECHNOLOGIES FOR SELF‐MANAGEMENT ENHANCEMENT

This paper contributes to the debate on the patient–doctor relationship, by focussing on a specific chronic disease, type 1 diabetes (T1D), with the aim to highlight the fruitfulness of a relational approach. T1D is a metabolic disease characterised by absolute insulin deficiency and is treated with lifelong insulin therapy (ADA, 2019). In order to prevent possible complications, an intensive self‐management is required (Daneman, 2006).

Even though self‐management still lacks a universal definition (Ellis et al., 2017), in this paper we find Audulv's (2013) contribution to be particularly useful, because he described it as the set of strategies undertaken by people with chronic conditions in order to control disease, promote health and live well with illness. This makes it ‘a complex developmental process that takes place within the context of a disease trajectory, a health‐care culture and a uniquely meaningful life’ (Thorne et al., 2003: 1349). Accordingly, self‐management is a ‘total life experience’ (Price, 1993), which requires the individual to adapt to fickle and unique life situations and contexts (Audulv, 2013). Indeed, diabetes is a tricky disease, in which ‘something unexpected may always happen’ (Mol, 2008: 91). This demands an ‘attentiveness’ (Mol, 2008) about daily practices including glycaemic monitoring, carb and insulin intake computation, and lifestyle behaviours, which relies on personal logic, experiences (Minet et al., 2011) and lay expertise (Storni, 2015). However, self‐management is not a stand‐alone procedure in which the single patient acts autonomously. Rather it recalls the idea of ‘logic of care’, by which patients are active in dynamic shared doctoring: this entails experimenting, attuning to one another and tinkering together, as well as exchanging experiences, knowledge, suggestions and words of comfort (Mol, 2008).

Diabetes self‐management has been enhanced by increasing technological innovation. Nowadays, the two main therapeutic devices (TDs) are those for continuous subcutaneous insulin infusion and those for self‐monitoring of blood glucose (ADA, 2019). Insulin pumps, for continuous subcutaneous insulin infusion, are small, portable, insulin injection devices. The latest pumps have advanced dose calculation software that can simplify daily self‐care, for example by recommending a bolus dose based on planned carbohydrate intake, blood glucose level, carbohydrate ratio, correction/sensitivity factor and also by considering the amount of active insulin in the body resulting from earlier boluses (Horwitz & Klonoff, 2017). For self‐monitoring of blood glucose, patients may use either real‐time continuous glucose monitoring or flash (or, intermittently scanned continuous glucose monitoring) sensors. They contribute to increase the number of daily measurements, which are important to set up the insulin intake. Moreover, by displaying the trend arrows, they can potentially support interventions with more accuracy (Galindo & Aleppo, 2020). Patients may choose to use just one or both of them. The combination of interoperable sensors and pumps may form sensor‐augmented insulin pumps (SAP), and a hybrid closed‐loop insulin delivery system, which is a step towards artificial pancreases. This further improves self‐management, even though patients must check their devices, as they do not work completely autonomously (Galindo & Aleppo, 2020; Taleb et al., 2021).

These devices affect self‐management in many ways, albeit not deterministically. For instance, these TDs allow huge data acquisition that potentially can be handled first and foremost by patients by allowing the practice of ‘clinical self‐tracking’. This is considered to be ‘part of a relationship that defines reciprocal duties, expectations and roles’ (Piras & Miele, 2017: 51). Therefore, all of this may, in turn, affect the doctor–patient relationship, as highlighted by a body of literature.

## THE PATIENT–DOCTOR RELATIONSHIP IN THE DIGITAL SOCIETY

The sociological debate on the patient–doctor relationship is, by and large, summarised by the juxtaposition between two opposite poles. On the one hand, we have the doctor‐centred model, which is well represented by the conceptualisation of an asymmetrical patient–doctor relationship (Parsons, 1951). In a nutshell, Parsons' paternalistic sick role provided an authoritative physician and a passive and compliant patient (Crossley, 1998). This theory was criticised, for example, because it failed to explain diseases with longer temporal horizons, such as chronic conditions (Crossley, 1998). Yet, some scholars stressed the enduring power of the medical profession and its authority as a ‘remarkable persistence of asymmetry’ (Murdoch et al., 2020; Pilnick & Dingwall, 2011: 1374), therefore highlighting the contemporaneity of Parsons' conceptualisation (Timmermans & Tietbohl, 2018).

On the other hand, we have the patient‐centred model. It took place by the end of the 20th century, as a consequence of some contextual changes (Heritage & Maynard, 2006; Nettleton, 2013), and in relation to the emergence of a consumerist approach in health care (Fraser et al., 2020). This new framework propelled a move from professional dominance to countervailing powers (Heritage & Maynard, 2006), or, in other terms, from patient compliance to a less authoritarian form of patient concordance (Nettleton, 2013). In such a context, the concept of patient‐centredness arose in order to promote a more ‘egalitarian’ idea of the patient–doctor relationship (Fraser et al., 2020; Mead & Bower, 2000; Timmermans & Tietbohl, 2018). The basic idea was that patients are not passive recipients, but active subjects involved in health‐care processes (Nettleton, 2013). However, some more drastic proposals also arose: van Olmen et al. (2011), for example, proposed the concept of ‘full self‐management’ as an approach that maximises patients' autonomy and independence.

This debate has been further fostered by the advancement of health‐care technologies. This field is dominated by the dichotomy between techno‐critics and tecno‐utopians (Lupton, 2016). Among the latter, some scholars (Swan, 2013; Topol, 2015) suggested that the revolutionary potential of technologies is capable of driving a paradigm shift from Parsonsian asymmetry to new assets, variously described as empowerment, active patient, expert patient and so on (Greener, 2008). To summarise, technologies magnify the ‘most vaunted of human commodities: choice, understanding, consciousness and freedom’ (Swan, 2013: 96), which conceptually push self‐management to its highest point. Accordingly, the massive data acquisition brought about by sensors and related apps should lead to a new data‐driven strategy and to the achievement of personalised and proactive self‐management. Among techno‐critics, Lupton (2014) highlighted the extension of the panoptical gaze of health professionals by means of technologies: since they create more ways of entering into people's private and intimate worlds, the role and power of physicians results to be strengthened. Another issue is the ambivalence often showed by patients as to whether they want to participate in their clinical decision‐making in addition to wanting to trust and rely on their doctors, and to be guided by them in medical decision‐making (MacArtney et al., 2020; Nettleton, 2013). In these contexts, scholars tend to stress the uncertain affective and digital atmospheres triggered by devices that are worn constantly, such as pumps or sensors, which might stimulate both sentiments of happiness or apprehension (Lupton, 2017).

The juxtaposition, outlined above, between doctor‐ and patient‐centredness seems to suggest ‘substantialist’ (Emirbayer, 1997), static and reified (Kaspersen & Gabriel, 2008) conceptualisations of power, which might be conceived as ‘something that one owns’ (Elias, 2008: 136): either the power is held by physicians, or by patients. Actually, some authors stressed more nuanced patient–doctor frameworks that go beyond such static representations. For example, the dynamic tensions underlying the practice of goal‐setting have been highlighted (Murdoch et al., 2020); or the ongoing conflict over power and control (Greenfield et al., 2012); or the ambivalence between logics of ‘being cared for’, or even ‘strongly guided’, and empowerment (Greener, 2008; MacArtney et al., 2020; Nettleton, 2013). In line with this more nuanced and dynamic analysis, we think that a relational perspective, which focuses on processes and flows of action (Powell & Dépelteau, 2013), as well as on transaction (Emirbayer, 1997), could be helpful in describing actual day‐to‐day patient–doctor relationships. As a matter of fact, it allows the idea to be overcome that both parties of this relationship are fixed, unchanging and independent entities. In contrast, it helps to stress dynamic, unfolding and ongoing processes (Emirbayer, 1997). The very idea of ‘centredness’ may be misleading, as actually there is no centre but rather an equilibrium or a balance. A vast dissertation about the relational ‘turn’ (Powell & Dépelteau, 2013) is beyond the scope of this work. Following previous studies (e.g. Brown et al., 2015), Elias' contribution seems to be particularly useful because he considers social life as a process of interdependent and interweaving social relationships (Kaspersen & Gabriel, 2008), in which the concept of figuration becomes paramount. For Elias (1978), human beings are *homines aperti* with a greater or lesser degree of relative, but not absolute, autonomy and dependency with other *homines aperti* (van Krieken, 2001). As highlighted by Dunning and Hughes (2013), humans are bound to others by fluid ties of interdependencies. Such interdependencies form dynamic figurations among people, which are described as ‘shifting networks of people with fluctuating, asymmetrical power balances’ (Dunning & Hughes, 2013: 52, Elias, 1978). In this sense, far from being ‘an isolated object in a state of rest’, power ‘is an attribute of relationships’ (Elias, 1978: 116); a ‘structural characteristic of human relationships’ (Elias, 1978: 74) since it lies in the interdependencies of people (Dunning & Hughes, 2013).

For these reasons, Elias' contribution helps to investigate in greater depth the doctor–patient relationship by allowing patients' possible behaviours and choices to be highlighted as the outcome of interdependent figurations and power balances. Consistently with this aim and theoretical background, we adopt Hirschman's exit‐voice model (1970) with a relational lens: the purpose is to understand how patients manage their satisfaction and dissatisfaction about the received care within interdependent figurations that are characterised by technology (TDs) and intensive self‐management requirements.

In Hirschman's theory, voice is defined as any conceivable strategy that changes an objectionable state of the art, with the aim of improving the situation (Hirschman, 1970). An effective voice requires at least three aspects (Pickard et al., 2006): users must be confident of being able to influence decision makers; there must be channels for communicating users' demands, such as in our case, the possibility for patients to express discontent directly to their diabetologists, or to access the Diabetes Centres through the public relations office; ‘voicing’ users cannot face penalties which might limit this option, such as being given worse treatment or being dismissed by doctors or Diabetes Centres. On the contrary, exit means that users abandon an organisation, ceasing to turn to it to receive their services without giving providers any feedback. For it to be a feasible option, other providers must exist and be reachable by exiting users. In the case hereby presented, the Italian National Health Service allows disappointed patients, aiming to change their current providers, to choose among all the existing Diabetes Centres, even those in other Regions. Of course, for the purposes of this paper, we only intend ‘active’ exit, namely when patients intentionally choose another professional, excluding the ‘passive’ exit, that is patients who are assigned to another diabetologist because they have moved (Vengberg et al., 2019). The third concept in Hirschman's archetype is that of loyalty. It is defined as a particular attachment to an organisation which supports their voice and lowers the tendency to exit. This is a key concept in the interplay between voice and exit, because without it, exit would become a costless option (Hirschman, 1970).

## METHODS

### Regional focus and selection of participants

This study was conducted in 2018 in one Italian Region, Emilia‐Romagna, which is considered top‐ranked in terms of the quality of health‐care services. This allowed us to override possible territorial differences due to the federalist framework of the Italian National Health Service (INHS) that in turn reflects different TD availability and requirement eligibility for accessing TDs. However, it is useful to remember that INHS guarantees patient free choice, so that they can receive their treatment from any health‐care facility (in our case Diabetes Centres, which are located inside hospitals) either within or outside regional boundaries (Lo Scalzo et al., 2009).

A qualitative approach was considered appropriate to our purpose to study in depth the relationship between physicians and patients and to understand possible strategies and motivations for patients to manage dissatisfaction. As TDs are only given free of charge to T1D in line with regional guidelines, we opted to recruit two kinds of actors: people with T1D and diabetologists. The latter, in fact, are by and large considered the most important professional point of reference in the Italian framework of diabetes care (AMD‐SID, 2018). These two kinds of actors were recruited through local patient and professional associations. For people with T1D, we were given a list of possible participants by patient associations; we then chose our respondents from that list according to our criteria. In contrast, establishing direct contacts with diabetologists was difficult for several reasons: for example workload; low propensity to pay attention to emails which are not strictly related to their professional tasks; difficulty to reach them on the phone or during working hours. This made doctors akin to relatively closed populations/élites (Cohen & Arieli, 2011). So, in this case, we opted to use a snowball technique as the most effective method for recruiting a sufficient number of diabetologists who met the study criteria. Overall, we recruited diabetologists through professional associations (3), patient associations (4), direct contact (2) and by using informal contacts with respondents gatekeepers (11).

The distinctive eligibility criterion was the use of at least one TD (for people with T1D) or caring patients who use TDs (for doctors). Then, the selection aimed to recruit participants of different ages, diagnostic timings (or timing since qualification, for doctors), geographical distribution within the Region, and both women and men.

We conducted 67 semi‐structured interviews (King, 2004): 47 with people with T1D and 20 with diabetologists. As no new information was emerging from both subgroups of respondents, we judged that the principle of saturation had been fulfilled (Edwards & Holland, 2013). Interviews were conducted by following some main topics, with the freedom to vary, skip or examine some of these issues in greater depth according to the personal narration of each respondent. The topics aimed to investigate the kind of TD adopted (when it was adopted, who proposed its use etc.); whether and how such TD/s supported and improved one's own self‐management; and then the main part focussed on the narration of the patient–doctor figuration (including issues such as the duration of the relationship; possible changes in the relationship after TD adoption; whether and how data produced by TDs were managed, analysed and discussed; the degree of involvement in such analysis and in the overall therapeutic decision‐making; patients' satisfaction or dissatisfaction with any aspect related to the care received and consequent behaviours; possible debate or even disagreement and arguments; whether patients had chosen to change diabetologist or Diabetes Centre for any reason; etc.).

The interviews were conducted from February to September 2018, in times and places that were convenient for participants, either face to face or by telephone. In both cases, all the interviews were audiotape‐recorded with the consent of the participants and subsequently transcribed verbatim. All data were rendered anonymous, and confidence was respected in all cases. All participants were previously provided with an information leaflet and a consent form, with which confidentiality and anonymity were guaranteed (Edwards & Holland, 2013; King, 2004). The study was approved by the Department of Sociology and Business Law of the University of Bologna.

### Analysis method

Data examination was performed through a ‘directed approach’ to content analysis, because our goal was to ‘validate or extend conceptually a theoretical framework or theory’ (Hsieh & Shannon, 2005), namely Hirschman's framework. Consequently, the exit‐voice model was used as the initial framework to identify the relevant information emerging from the respondents. The interviews were processed and coded with the aid of NVivo 12. We developed the coding process by following Hsieh and Shannon's (2005) description. An initial close line‐by‐line reading of each interview permitted the detection of all passages related to the doctor–patient relationship, which were given a first label (creating nodes); further reading allowed us to better contextualise these excerpts into the whole context of each interview and, then, we were able to verify, confirm, merge or change the names of the nodes. Accordingly, a final list of nodes was produced. Next, such nodes underwent a further close reading and then were coded according to Hirschman's model (exit, voice, loyalty). The excerpts‐nodes that did not fit into such a threefold scheme underwent further examination and were then grouped into a new category that we named ‘resignation’.

## MAIN FINDINGS

The main characteristics of participants are outlined in Tables 1 and 2.

**TABLE 1:** Summary of people with diabetes

Age (mean, range)	45.04 (19–72)	Pumps^a^		Sensors (both types)^a^	
		Used	Not used	Used	Not used
18–24	8	6	2	6	2
25–34	9	5	4	7	2
35–44	4	3	1	3	1
45–54	10	5	5	9	1
55–64	9	3	6	8	1
65–70	4	0	4	4	0
71+	3	0	3	2	1
Gender					
Men	16	7	9	12	4
Women	31	15	16	27	4
Duration of disease (years)					
<9	4	1	3	3	1
10–15	12	9	3	11	1
16–20	9	4	5	5	4
21–30	6	3	2	3	2
31–40	9	1	8	8	0
41+	7	3	4	7	0

^a^ Some patients use both pumps and sensors.

**TABLE 2:** Summary of diabetologists

Age (mean, range)	52.15 (36–67)
35–44	7
45–54	2
55–64	9
65–70	2
Gender	
Men	7
Women	13
Time since qualification (years)	
<10	5
11–20	5
21–30	7
31+	3

Findings show that interdependent patient–doctor figurations can produce four possible outcomes: exit, voice, loyalty and resignation. These behaviours are fluid because patterns depend on the evolution of the figurations that might be built among the transactors. Most of the participants (28) experienced just one behaviour, but fourteen participants experienced two behaviours, while five experienced three outcomes. The mechanism behind the possible different choices is the everchanging power balance in such figurations. Both actors tend to recognise more balanced transactions than those suggested in patient‐ or doctor‐centredness scenarios. In this case, patients' substantial requirement for self‐management and the use of TDs seem to be at the centre of this power balance, as the following excerpt suggests:

the patient who uses new technologies is more knowledgeable and smarter; so, let's say, during the visit, well, sometimes it looks like we're even. I mean, we're experts, but patients have become experts as well; therefore, maybe beyond formulas and rules of insulin doses, sometimes it's the patient to tell us “no, I'm not fine with three doses here, because my glycaemia soars,” and then they show you their monitoring. At the end of the day, it's a discussion among peers. (diab‐3, woman, 37, Time‐since‐Qualification <10yrs)

This confirms that diabetes self‐management is neither a matter of ‘attending medical prescriptions’ (Storni, 2015: 1447), nor of being independent full self‐managers (van Olmen et al., 2011), but rather an interdependency which produces a ‘shared doctoring’ (Mol, 2008). Besides, it also goes beyond experiential knowledge and lay expertise (Maslen & Lupton, 2019). Indeed, by means of clinical self‐tracking practice (Piras & Miele, 2017), TDs offer users real‐time data that allow them to get to know in greater depth how their own bodies and diabetes are working on a daily basis. This gives patients a certain degree of power to negotiate different forms of interdependent figurations with diabetologists. Undeniably, TDs may help to support self‐management, as explained by an interviewee:

some months ago, I realised I had very low glycaemia. Then, I downloaded my data, I checked them and I saw when my glycaemia went down. I detected a trend (…) and I made correction right there. So, yes, it's actually useful! I can even change my therapy on my own, thanks to these data. But, then, analysing them all, no, I never do it. (pat‐27, woman, 26, Duration‐of‐Disease 16‐20yrs, using pump and ‘continuous’ sensor)

So, a high level of self‐management skills and good knowledge and competence regarding TDs are important resources for power balances in favour of patients. However, this does not lead straightforwardly to the figure of a doctorless T1D patient (Topol, 2015), as the latter rather tends to combine autonomy and an attitude to relying on physicians, or caring (Greener, 2008; Nettleton, 2013). In other words, T1D patients have the desire for a ‘productive relationship’ with their doctors (Fraser et al., 2020). It is exemplified by this excerpt:

I manage it a while, but I need doctors as reference points; I can't do it on my own, this is not a disease which you can manage alone. You need doctors, and even good ones, because they must be able to tell you “no, there's a mistake here,” or “no, here you have to do this”. (pat‐47, woman, 53, Duration‐of‐Disease 10‐15yrs, using pump and ‘continuous’ sensor)

Increasing patient competence both on clinical and technological grounds within a framework of self‐management and shared doctoring creates high expectations regarding the figurations with their diabetologists. They are required to have clinical competence, technological skills and also the capacity to share the doctoring, which entails relational traits such as exchanging knowledge, suggestions and experiences, as well as giving words of comfort (Mol, 2008) and empathy (Brown et al., 2015; MacArtney et al., 2020). These requirements reject asymmetrical and paternalistic relationships, as well as seeing patients as independent figures sublimated by concepts of empowerment and patient‐centredness. Rather, they help shape different interdependent figurations, which in turn may facilitate specific behaviours.

### Exit

Eleven patients talked about exit events. Sometimes, disagreements (related to interpersonal skills or therapeutic patterns) preceded the subsequent choice of changing diabetologist. Exit might mean either seeking another Diabetes Centre (if the patient desires so and as permitted by the INHS), or another diabetologist within the same centre, when the organisation of the centre itself allows it. In some cases, change is aimed at seeking a leading Diabetes Centre, while in most cases it is due to a particularly disappointing figuration experienced with a specific team.

The following excerpt summarises the interrelation of all the three dimensions, as the basis for an exit strategy:

(…) the fact is that we realise that there are few diabetologists who are competent about technologies (…). I changed the diabetologist. (…) I tell you that my life has completely changed since! He was old‐fashioned, arrogant, overbearing, what else? Mean! It's been really hard, also because the patient feels a bit of awe in front of the diabetologist; because you know, he studied, not me; and then there's a trust that must be placed well in them. And sometimes, it's difficult to realise that it was misplaced (…) You trust him, but, well… you think a bit naively that he knows everything, you don't bring his competence into question. Instead, I've got 38 years of ill‐compensated diabetes. At the beginning there were no devices, then the diabetologist is lacking [meaning his clinical and technological competence]… and I've had for many years 9 of Hgb. (pat‐1, woman, 52, Duration‐of‐Disease 31‐40yrs, using ‘flash’ sensor)

This excerpt confirms the importance of trust for the quality of the figuration, because it affects self‐management, among other things (White et al., 2013). Moreover, it is intertwined with clinical‐technical competence and interpersonal skills, such as exchange and empathy (Brown et al., 2015; MacArtney et al., 2020; Mol, 2008; Peek et al., 2013). Besides, we also see the relevance of technological skills, as patients have bridged the gap with the diabetologists, or even know more than them, and they demand these TDs (Bos et al., 2008). Peek et al. (2013) highlighted that trust can affect preferences in different kinds of roles in decision‐making process, such as passive, shared or autonomous. In our case, as INHS allows it, mistrust and dissatisfaction for a not‐shared doctoring figuration may change the power balance in a way that in the end led to exit. Nevertheless, exit does not imply getting out of the system, but seeking another doctor, with whom the desired satisfying shared doctoring figuration can be built, whose importance was suggested by Fraser et al. (2020). Consequently, exit does not entail autonomy, or a kind of patient‐centredness that consults doctors on demand (van Olmen et al., 2011), but the need to build a new figuration with another transactor, which will be characterised by another power balance.

### Voice

Voice was the least frequent behaviour among our participants: only seven interviewees declared having argued with a doctor. One explanation could be a refrain from voicing, because patients still feel deference towards their doctors, even though a recent research showed the decline of this feeling (Brown et al., 2015). On the whole, in this research it seems that most people genuinely consider their figurations with their diabetologists in a positive light. The following excerpt exemplifies an event of voice that aimed to receive a certain kind and brand of pump, rather than another one that was proposed by the diabetologist. It summarises the relevance of technological skills on both sides (patient and diabetologist), as well as the need to rely on doctors and on the health‐care system (Nettleton, 2013), but not in a supine asymmetrical way. Besides, technological suggestion and acceptance is the example of interdependent transactors, as we can read in the narration of this interviewee. Indeed, as a dance (Elias, 1978), both transactors are only relatively independent, and no one has absolute power over the other: the doctor suggested a device, but the patient rejected it, because it was complicated; on the other hand, diabetologists are the gatekeepers for giving TDs free of charge.

That time I got really angry, I mean… She dug her heels in about giving me a type of pump (…), that was too complicated for me. I was used to my old pump, that I could manage in the dark, instead she wanted to give me that thing. I gave it back to her and I told her: ‘no…I don't want that, I want my [xy]’. [The diabetologist said:] ‘eh, but we have to wait a lot to get the [xy]’. (…) So, I called the corporate representative and told him: ‘for a research my association is doing, I'd like to know how much we must wait for having [xy]’. (…) And he told me: ‘ah, I'll bring it to the Department in 2‐3 days; better, I'll be in [town of the Region] on Monday, if the doctor wants…’. (…) He figured the business out. (…) Then, I went [to the Department to meet the diabetologist, but] she said ‘I haven't time for chatting now’, and I said bluntly: ‘look, this is the code for my [xy]; please call [the company] because it arrives in 2‐3 days’. I pissed her off a bit. (pat‐7, woman, 71, Duration‐of‐Disease 31‐40yrs, using ‘flash’ sensor and pump)

### Loyalty

Forty participants shared feelings of loyalty towards their diabetologists and/or team, making it the most highly adopted behaviour among our participants. Generally speaking, loyalty seems to be more likely when patients perceive being and feeling well:

I've always had good levels of Hgb, well, I mean… I'm quite ok with my diabetes. Even if I'm not ok, eheh, I'm quite ok. Hence, well, in my opinion, my Diabetes Centre has been treating me properly. (pat‐37, woman, 54, Duration‐of‐Disease 21‐30yrs, uses flash sensor)

Loyalty emerged as an attachment to the Diabetes Centre or the doctor, which is spurred by the experience of satisfaction, whose importance was already highlighted by De Rosis and Barsanti (2016). This finding, besides, is not surprising given the standard of quality that ranks Emilia‐Romagna among the best Regions of INHS.

The concept of loyalty, in this research, has two features, which are in line with Hirschman (1970). First, it is vigilant and potentially ready for voicing, rather than exiting:

I don't know, if we speak about a particular procedure that's used in Modena but not here, let's say, I turn to the confederation, in addition to the doctor; we've got a confederation of all the patients' associations of our Region (…) I mean, [we must] turn to doctors, but also ask for support from community associations. (pat‐28, woman, 48, Duration‐of‐Disease 21‐30yrs, uses pump and ‘continuous’ sensor)

Second, it does not mean supine compliance or obedience:

I think that, at the end of the day, the diabetologist must give you a tip about how to do it and teach you, and then you have to manage yourself. (…) sure, if you stray, he must help get you back in line; so, he's got to be good at training you how to self‐check because it goes up and down every day (…). No, it doesn't get fixed on its own and you must not call the diabetologist over and over again. (pat‐13, man, 66, Duration‐of‐Disease 31‐40yrs, uses ‘flash’ sensor)

The latest excerpt stresses the importance of self‐management as a developmental process according to the everchanging complexity of diabetes (Thorne et al., 2003); besides, it does not suggest passivity, but a shared doctoring (Audulv, 2013; Mol, 2008).

The following participant expresses the behaviour of loyalty, in which the importance of self‐management and of the chance of using TDs merge together in a patient–doctor figuration that seems to be extremely balanced, and in which interdependency is well described, above all regarding TDs. Therefore, TDs do not foster autonomy (Swan, 2013), but promote new figurations in which a balance between caring and empowering is relevant (Greener, 2008):

I always relied on our National Health Service (…), I've always been treated by a diabetologist of the Diabetes Centre of my town, without looking elsewhere (…). This is also because I go back to what I said before: you can surely choose to go anywhere (…) But, referring to glycaemic control, as I said before, with this chronic disease, (…) the self‐management is important; then, the professional, your reference, is important of course, but it's what you do for yourself that helps you feel better or not. And this chance is related to the available devices that [the Diabetes Centre] gives to you. (pat‐28, woman, 48, Duration‐of‐Disease 21‐30yrs, uses pump and ‘continuous’ sensor)

### Resignation

Findings suggest a possible fourth behaviour as the consequence of different causes, such as the feeling of disappointment and incapacity of changing things, or the belief that things work in the same way everywhere else. These elements fail to nurture the spirit of loyalty and, even though they increase the cost of an exit, they also deprive the patient of their voice. On the whole, there is little trace of any enthusiastic adhesion or feelings of belonging to the INHS and/or a specific doctor/team, or at least of the choice to stay with the expectation that matters will be improved (Hirschman, 1970). The consequence is that the patient maintains the relationship in a situation that can instead be framed as a complaint‐free (no voice) resignation (no exit). All this notwithstanding, one could choose to be treated by any facility in the whole country, as permitted by the INHS. Thirteen participants told life stories frameable within this category.

The following excerpt offers a valid example of resignation, as well as of the fluidity of the behaviours, as the interviewee had opted for an exit strategy before choosing to be treated by the doctor, whom she speaks about in this passage:

I moved to another diabetologist, with whom I stayed until he retired; he was a problematic individual, in my opinion (…); well, I kept going to him, because I didn't want to change town to change diabetologist, or maybe just because I thought that this was the standard; but, often after the visit I cried, and this must not happen! And I realised it after he retired, when I started to be treated by another diabetologist, who doesn't think that he's God, eheh; he manages to be humble and simple; and I can have a relaxed conversation with him, that I like and it helps me a lot. He never thinks that he can solve problems, but he proposes his view and then we talk about it. (…) For instance, a big flaw of that [previous diabetologist] was that he focused on theory, and when I told him “it doesn't work for me,” giving him practical examples, he dismissed me by saying “but, you're different.” But no, it's the theory that has to be changed if I tell you experiences that don't confirm it; that's paramount, otherwise, the theory remains far from patients, and it crushes patients. Yes, this was really frustrating for me. (pat‐33, woman, 46, Duration‐of‐Disease 31‐40yrs, uses ‘continuous’ sensor)

In this case, the respondent did not refer to TDs, but stresses the relevance for shared doctoring. By highlighting the practice of self‐management as a process of interdependence with her diabetologist, she basically compared two opposite figurations: the first characterised by a strong asymmetry of power and paternalism; the second more even and balanced and subsequently more satisfying, albeit still interdependent. To conclude, the excerpt pinpoints that patients with T1D cannot face their condition alone but need to stay in a figuration.

## DISCUSSION

By investigating the patient–doctor relationship in the milieu of diabetes care, this paper highlighted its relational and interdependent characteristics, which form specific dynamic figurations. Patients' management of satisfaction and/or dissatisfaction takes place within such figurations and allows them to enact four different behaviours. We also showed that these behaviours are not deterministic, but they are the outcomes of the kind and quality of particular patient–doctor figurations.

Therefore, our findings do not support patient‐centred approaches that aim to highlight characteristics such as autonomy, independence and ‘full self‐management’ (Swan, 2013; Topol, 2015; van Olmen et al., 2011). For instance, Armstrong (2014) postulated the emergence of a ‘new patient identity’ in connection with the increasing relevance of patient autonomy and her/his centredness: namely, a patient who exercises agency in terms of preparedness, vigilance and capability of making autonomous choices. However, in this paper, respondents who opted for loyalty and resignation indicated a much more tangled relationship, based on interdependency. Besides, we show that even when choosing the exit strategy, which is the most disruptive scenario, patients do not seek autonomy and independence, but on the contrary a new diabetologist to trust and with whom to build a new figuration. These findings are more in line with Fraser et al. (2020), who described patients' desire to have ‘an honest and productive relationship with a professional health practitioner’. So, we could say that ‘patient agency’ is exercised to a greater extent within a patient–doctor figuration rather than in autonomous and independent assets.

On the other hand, our findings do not support doctor‐centred or traditional asymmetrical frameworks either (Murdoch et al., 2020; Parsons, 1951; Pilnick & Dingwall, 2011). In particular, Pilnick and Dingwall (2011: 1381) postulated that the persistence of asymmetry is embedded within a wider functionality of the institution of medicine in society. We also reported the existence of asymmetrical figurations, as described in the excerpts related to resignation, for example. However, both ‘voice’ and above all ‘exit’ allow us to state that asymmetry is not static, but a dynamic and fluid characteristic of figuration which may entail different scenarios.

Both these static and substantialist ideas of relationship and power, centred and focussed only on one actor, are incapable of explaining actual nuances in the patient–doctor relationship. On the contrary, our findings support the idea that interdependent and figurational perspectives seem to be more consistent and useful in shedding further light on the ambivalence existing in doctor–patient relationships. Some of these contradictions have been pinpointed in the sociological literature. For instance, Greener (2008) stressed the co‐existence of the logics of caring and empowering, whereas Phillips and Scheffmann‐Petersen (2020) stressed the co‐existence of both models of doctor‐ and patient‐centredness and considered the relationship as the unstable product of dialogic meaning‐making processes that are inherently complex and full of tensions. By the same token, MacArtney (2020: 855) underlined the ‘limitation of dichotomised framings of the patient–doctor relationship’, by stressing both the asymmetries of knowledge and social capital, on one side, and patients' empowered choices and individualised care, on the other side. Our findings corroborate the need to go beyond ‘dichotomised framings’. As a matter of fact, by using a figurational framework, we could explain that such ambivalences mentioned above are shaped by interdependent and dynamic figurations that rely on unstable power balances. In turn, this unstable equilibrium can lead people with T1D to manage their personal (dis)satisfaction with doctors by choosing one among loyalty, voice, exit, resignation. As purported by Murdoch et al. (2020), patients and doctors exercise their own power in a subtle interactional tug of war, in which there arises ‘a covert conflict over power and control, where each party plays its own cards, striving to preserve autonomy and control’ (Greenfield et al., 2012: 1208). Consistent with this, we describe power as not being a static possession, as well as transactors not being pre‐determinedly fixed and independent entities. In this way, we can state that patient agency is interdependent with doctor agency: accordingly, consultations and figurations take shape in ongoing processes and unstable equilibriums that rely on dynamic and fluid power balances among transactors.

Power balance is the mechanism that explains how people with T1D manage their own (dis)satisfaction within patient–doctor figurations. Our respondents showed that this balance is affected by three relevant variables: one basic interpersonal dimension and two technical skills. The first one, the interpersonal dimension, allows shared doctoring (Mol, 2008). Our findings show the importance of this issue, according to which doctors are expected to be empathetic and open (Brown et al., 2015; MacArtney et al., 2020), as this can enhance patients' satisfaction. However, we also show how enacting such interpersonal attitude does not exempt diabetologists from deciding (MacArtney et al., 2020) about, for example, therapy or TD prescriptions. The technical dimensions entail clinical‐therapeutic skills and technological skills. Our findings show that a certain degree of expertise attained over time by people with T1D reduces the sharp epistemic asymmetry, which relies on a knowledge gap (Montenegro & Dori‐Hacohen, 2020), sustaining self‐management tasks. They also allow us to go beyond the concepts of experiential knowledge and lay expertise (Maslen & Lupton, 2019). Indeed, our study described that, by using TDs, people with T1D ‘may also have unique epistemic access (knowledge), and even epistemic primacy and authority, over their day‐to‐day diabetes‐related behaviours’ (Montenegro & Dori‐Hacohen, 2020: 2). This happens when, as demonstrated, people with T1D can use and (self)manage data produced by TDs to act on their own therapies through clinical self‐tracking (Piras & Miele, 2017). This contributes to make figurations further ‘complicated by potentially competing knowledge between physicians and patients’ (Montenegro & Dori‐Hacohen, 2020: 2). Of course, these variables, while affecting power balances, do not entail deterministically one specific outcome, as it will evolve only within each particular figuration.

## LIMITATIONS

This study has some limitations. First, the recruitment of a sufficient number of diabetologists was a difficult and challenging operation. Some characteristics, discussed above, rendered them akin to closed élites. Besides, we had to take into account the time constraints regarding the research (the fellowship was programmed to last only one year). Therefore, in such a context, snowball sampling was considered to be the most efficient method to add further participants, even though several shortcomings and biases make it only a second‐best methodology (Cohen & Arieli, 2011). Second, in this study we checked, but did not, find specific differences according to personal variables (gender, age, duration of disease): their potential relevance should be analysed in future research with bigger samples. Third, the study was conducted in only one Region: future studies should investigate at a national, if not international, level, in order to check how different health‐care systems might shape both figurations and outcomes. Last, in our sample, basically all respondents were using at least one TD; given the low percentage of their use (e.g. Bruttomesso et al., 2015), our sample represents a niche, and this might have affected our findings. Nonetheless, this study allows light to be shed on a particular kind of patient–doctor figuration, when high levels of self‐management are required to patients and simultaneously when TDs are used for sustaining the daily patient's management. Given that the relevance of both chronic conditions, such as diabetes, and technological development are going to increase, these findings seem to be useful.

## CONCLUSION

This study suggests that relational perspectives are better equipped for analysing patient–doctor relationships, by describing them as interdependent figurations. Indeed, this seems to be important in the field of chronic conditions, where such transactions last for the entire lifetime of patients. Besides, this approach seems to be particularly promising when considering milieus in which both self‐management and TDs are becoming increasingly important. In such figurations, smarter and knowledgeable patients have high demands about three paramount variables that affect one's own self‐management, as well as the figuration itself: interpersonal, clinical‐therapeutic and technological skills. They are the three cornerstones for dynamic balances of power and also for the figurations themselves. The power balance may help explain certain behaviours adopted by patients, which are naturally fluid, and can be framed according to a fourfold exit‐voice model, which stems from Hirschman's idealtype (1970). Even though loyalty prevails, when one or more of the abovementioned cornerstones falter, exit, voice or resignation might arise. Thus, the study contrasts both patient‐ and doctor‐centred models, above all in their drastic forms of doctorless full‐self‐manager patients (Swan, 2013; Topol, 2015; van Olmen et al., 2011), and asymmetrical patient compliance and passivity (Parsons, 1951). As a matter of fact, these models seem to be too static, and not suitable to stressing dynamic and interdependent transactions, which actually do exist and, at least in part, account for patients' different choices in their management of (dis)satisfaction.

Future research should broaden the understanding presented here by following two directions based on relational perspectives. On the one hand, by considering the role of social capital and different health‐care systems in contributing to shaping power balances and choices‐behaviours. On the other hand, by studying diseases characterised by lower degrees of self‐management requirements, and TD availability and use: this would allow further understanding as to whether this fourfold framework can be applied in general, or alternatively to assess the specific role of these two issues. Moreover, it would also facilitate understanding as to how the dynamic power balances may be shaped in such milieus.

## AUTHOR CONTRIBUTION

**Alberto Ardissone:** Conceptualization (lead); Data curation (lead); Formal analysis (lead); Investigation (lead); Methodology (lead); Writing‐original draft (lead); Writing‐review & editing (lead).

## Data Availability

The data that support the findings of this study are available on request from the corresponding author. The data are not publicly available due to privacy or ethical restrictions.
